# Répercussions psychosociales de la drépanocytose sur les parents d'enfants vivant à Kinshasa, République Démocratique du Congo: une étude qualitative

**DOI:** 10.11604/pamj.2014.19.5.2830

**Published:** 2014-09-02

**Authors:** Evariste Luboya, Jean-Christophe Bukasa Tshilonda, Mathilde Bothale Ekila, Michel Ntetani Aloni

**Affiliations:** 1 Institut Supérieur de Technique Médicale, Mbuji-Mayi, Kasaï Oriental, République Démocratique du Congo; 2 Département de Médecine Interne, Cliniques Universitaires de Kinshasa, Faculté de Médecine, Université de Kinshasa, Kinshasa, République Démocratique du Congo; 3 Division d'Hémato-oncologie et Néphrologie, Département de Pédiatrie, Cliniques Universitaires de Kinshasa, Faculté de Médecine, Université de Kinshasa, Kinshasa, République Démocratique du Congo

**Keywords:** Drépanocytose, répercussions psychologiques, enfant, Kinshasa, République Démocratique du Congo, sickle cell disease, psychosocial impact, children, Kinshasa, Democratic Republic of Congo

## Abstract

**Introduction:**

L'insuffisance des moyens de base pour le dépistage et la prise en charge de la socioculturel Africain. D'où la nécessité de réaliser un travail de mise en sens du vécu et des émotions en vue d'information et de soutien psychologique des familles des drépanocytaires. Cette étude a eu pour objectif d'identifier la nature des répercussions psychosociales de la drépanocytose chez les parents et chez les malades.

**Méthodes:**

Une approche qualitative a été utilisée. Des entretiens ont été menés auprès des parents et des patients drépanocytaires. Nos résultats ont fait l'objet d'une analyse thématique articulée sur les circonstances de découvertes de la maladie, les répercussions de la maladie et la perception de la prise en charge.

**Résultats:**

Nos interviews ont montré des répercussions psychosociales importantes chez les parents d'enfants drépanocytaires et de stigmatisation des difficultés d'insertion sociale et scolaire pour les enfants drépanocytaires. Ces derniers sont les grands oubliés des récits de parents. La prise en charge est uniquement médicale avec des ressources très limitées et aucun accompagnement psychologique de la famille n'est assuré.

**Conclusion:**

La prise en charge de cette maladie nécessite la mise en place d'une politique de prise en charge basée sur une approche globale de la maladie.

## Introduction

La drépanocytose est la maladie génétique la plus fréquente dans le monde. Les différentes migrations ont favorisé son expansion dans le monde [1]. Dans son rapport de 2006, l'OMS estime que 500 millions d'individus sont porteurs du trait drépanocytaire et qu'environ 50 millions d'individus vivent avec la maladie [2]. Chaque année, 300.000 enfants naissent avec la maladie dont les 2/3 en Afrique sub-saharienne [1].

En Afrique, la fréquence des porteurs du gène de la drépanocytose est variable et peut atteindre des prévalences de 40% dans certaines populations [3, 4]. En République Démocratique du Congo (RDC), les données épidémiologiques récentes ont montré qu'en période néonatale, 2% de nouveau-nés sont homozygotes pour la maladie et environ 40.000 naissances d′enfants drépanocytaires sont estimées chaque année, tandis que dans la population adulte le portage du trait s′élève à 25% et la forme homozygote affecte environ 2% des individus [5, 6]. Si ce chiffre est significatif au point de vue épidémiologique, la maladie reste peu connue avec pour conséquence une forte mortalité dans un pays à ressources limitées [7–9].

La drépanocytose est caractérisée par des crises douloureuses et des crises hématologiques exposant à un risque transfusionnel important et une forte susceptibilité aux infections. Ceci rend compte de la forte morbi-mortalité enregistrée chez les drépanocytaires et que 50 à 80% des enfants nés sur le continent africain n'atteindront pas l’âge de 5 ans [8, 9]. A côté de ces manifestations aiguës, la drépanocytose associe des complications chroniques dégénératives au niveau des organes [10, 11] et un retard staturo-pondéral et pubertaire [12, 13]. Elle entraîne des séjours fréquents à l'hôpital. Au Congo-Brazzaville, les crises douloureuses représentent la première cause d'hospitalisation [14].

La lutte contre la drépanocytose met à contribution plusieurs secteurs et les incidences en santé publique sont importantes dont les conséquences peuvent être évaluées par rapport à la mortalité infantile, particulièrement celle des moins de 5 ans. Dans la plupart des pays où la drépanocytose constitue une préoccupation majeure de santé publique, les moyens de base pour sa prise en charge sont restés insuffisants, le dépistage systématique de la maladie à la naissance n'est pas de pratique courante et le diagnostic est généralement posé tardivement [8, 9, 15].

Contrairement aux pays du Nord, les patients drépanocytaires en RDC ne bénéficient pas d'un suivi médical rigoureux et régulier à la recherche des complications organiques auxquelles expose la maladie [8, 9, 15–17]. En outre, les ressources humaines, financières, matérielles et les centres spécialisés dans la prise en charge de la drépanocytose et ses complications sont rares. La prise en charge reste quasiment uniquement médicale. Le système de santé est dominé par les programmes verticaux orientés vers des pathologies particulières (paludisme, tuberculose et VIH/SIDA), ceci désorganisant le système intégré des soins de santé. En outre, les drépanocytaires, à l′instar de l′ensemble de la population, vivent dans un système sans sécurité sociale.

En parallèle des soins médicaux, des études ont montré que le succès de la prise en charge de la maladie résidait dans une approche globale centrée sur le malade et sa famille [18]. La drépanocytose est une maladie chronique invalidante sujette à de nombreux tabous et de nombreuses stigmatisations dans nos sociétés Africaines pouvant aller de l'isolement et de la maltraitance du patient au divorce des parents [19–24].

Ce tableau alarmant du vécu de la maladie dans un contexte socioculturel Africain montre la nécessité de réaliser un travail d'information et de mise en sens du vécu et des émotions de familles des drépanocytaires. L'objectif de cette étude est d'identifier d'une part les répercussions psychosociales de la drépanocytose vécues par les parents à différentes étapes de la maladie: l'annonce du diagnostic, la prise en charge et après le décès; d'autre part, les besoins et les problèmes liés à la prise en charge des drépanocytaires auprès de ces parents. Pour être identifiées, ces différentes répercussions nécessitent un diagnostic communautaire en considérant que la santé et la qualité de vie sont intimement liées.

## Méthodes

La drépanocytose est lié à une représentation de la mort dans le contexte sub-saharien Les familles vivent dans l'incertitude face à la tragédie. Devant cette situation, la drépanocytose reste cachée de l'entourage familial; seuls les parents gèrent la situation même pendant la crise vaso-occlusive chez l'enfant. Compte tenu de la sensibilité des informations que nous recherchions, une approche qualitative a été utilisée afin de faire ressortir les répercussions psychosociales ainsi que les problèmes et les besoins liés à la prise en charge de la drépanocytose dans notre milieu d’étude. Elle est facilitée dans ce cadre par le processus du diagnostic communautaire qui permet à une communauté d'identifier elle-même ses problèmes, besoins et les ressources pour répondre à leurs problèmes.

Pour collecter les informations, nous avons eu recours aux entretiens individuels semi-structurés avec les parents et les tuteurs des enfants drépanocytaires à l'aide du guide d'entretien construit sur base de nos questions de recherche et nos objectifs. Les personnes interviewées ont été recrutées au Centre de Médecine Mixte et d'Anémie SS (CMMA SS) de Yolo à Kinshasa appelé aussi Mabanga, littéralement la « pierre » en lingala, la langue prédominante à Kinshasa et qui renvoie à la notion de la splénomégalie par la population. Au fil des contacts, la méthode dite « en boule de neige » nous a permis de recruter un échantillon16 parents. L'entrevue s'est déroulée dans la langue parlée par chaque participant, soit 1 entretien en français, et 15 en lingala (langue parlée couramment à Kinshasa). Nos résultats ont fait l'objet d'une analyse thématique articulée autour de quatre idées principales: (i) les circonstances de découvertes de la maladie, (ii) les répercussions de la maladie, (iii) la perception de la prise en charge, (iv) les besoins et les problèmes liés à la prise en charge et des solutions proposées.

## Résultats

### Caractéristiques des personnes interviewees

La population d’étude a été composée de 16 parents ou tuteurs d'enfants drépanocytaires que nous avons rencontrés. Parmi eux, 11 parents et tuteurs ont été inclus à l’étude pour des raisons de disponibilité et fiabilité des informations sur la prise en charge de la maladie. Ce petit échantillon s'explique par le fait que de nombreux parents n'ont pas voulu s'exprimer sur la drépanocytose, ils pensaient que parler de son enfant drépanocytaire, les amèneraient à se remémorer de mauvais souvenir de la maladie. Parmi les personnes rencontrées, les mères occupaient la première place (9/11), une grand-mère et un frère au malade drépanocytaire. Trois parents avaient déjà perdu au moins un enfant de suite de la drépanocytose. Parmi les drépanocytaires rencontrés avec leurs parents lors des entretiens, le plus âgé avait 28 ans d’âge et le moins âgé, 2 ans et demi.

### Circonstances de découverte de la maladie

Selon le contenu des entretiens de parents ou tuteurs d'enfants drépanocytaires suivis dans ce centre, dans la plupart de cas, la maladie a été découverte lors d'une hospitalisation, et les principaux motifs de consultation étaient principalement la fièvre, la pâleur et le syndrome pieds-mains. Pour certains de nos enquêtés, la consultation avait été recommandée par des personnes ayant des connaissances sur la prise en charge des malades.

### Parmi les entretiens, nous présentons deux récits sous forme de cas Clinique

« Lorsque nous étions à Luanda (Angola), à 3 mois d’âge, ma fille avait souvent de fortes fièvres à répétition avec des pleurs interminables jusqu’à l’âge de 6 mois. A 9 mois d’âge, j'avais soupçonné la malaria et j'ai commencé à lui donner la quinine mais l'enfant s'agitait de plus en plus. Ma grand-mère a pris l'enfant, et l'ayant regardé, a vu que l'enfant était trop pâle. Nous avons fait recourt à une infirmière qui nous a orienté à Kinshasa au centre Mabanga. De là, on a fait l’électrophorèse qui a révélé la maladie » (Madame R.). « A 1 an, on a remarqué le gonflement de deux pieds et les deux mais, on s'est demandé de quoi s'agissait-il? Nous sommes allés à l'hôpital, on a fait les examens et on nous a dit que l'enfant est SS à 100%. Nous nous sommes examiné aussi, moi sa mère je suis du groupe B et son père a le groupe O, donneur. Mais comment expliquer qu’étant de groupes différents qu'on donne un enfant SS? Et pourtant, nous allons au contrôle médical régulièrement » (Madame N). En résumé, l'analyse des récits des enquêtés révèlent que tous les parents des enfants drépanocytaires n'avaient pas les connaissances requises pour détecter des signes précurseurs de la drépanocytose. Ils assimilaient les symptômes présentés par leurs enfants avec ceux des maladies habituelles de la petite enfance (malaria, fièvres isolées, etc.). Les circonstances de la découverte de la maladie semblent être fortuites.

### Les répercussions de la drépanocytose

#### A l'annonce de la maladie

**Sentiment de panique:** l'image de la drépanocytose véhiculée dans les sociétés sub-Saharienne est celle d'une maladie honteuse, liée à la malédiction, à une punition de la nature pour une faute commise dans le passé dont l’évolution unique est le décès de l'enfant. La majorité des interviewés ont eu à connaître des drépanocytaires dans leur milieu ce qui explique qu'ils affichent un sentiment de panique. Ce sentiment est dû aux informations véhiculées dans la société: l'espérance de vie trop courte des enfants drépanocytaires. La maladie qui est chronique modifie le contexte familial. Les parents en général, et les femmes en particulier, pensent que cette découverte de la maladie pourrait mettre fin à leur mariage si les membres de la famille étaient au courant de cette situation.

**Angoisse et inquiétude:** en analysant les propos de nos enquêtés, il convient de signaler qu'aucune personne n'a pensé à la drépanocytose, bien que la prévalence de la maladie soit importante dans les populations congolaises et que dans la plupart de cas les signes annonciateurs étaient médicalement suggestifs de la maladie avant d'aller à l'hôpital. L'annonce de la maladie plonge les parents dans l'angoisse et l'inquiétude permanentes; angoisse due au fait que la maladie est chronique et létale dans les milieux à faibles ressources comme la RDC. Le sentiment d'inquiétude est engendré par le fait qu'ils imaginent leur enfant souffrir toute sa vie. Devant cette situation et sous la pression des belles-familles, ceux de l'homme particulièrement, le divorce est perçu comme la solution pour mettre fin à la malédiction. La femme étant perçue comme celle qui a transmis la maladie.

### Au cours de la vie de l'enfant

#### Chez l'enfant

Pour certains parents, les enfants n'ont pas de problèmes en dehors de crises. Ils mènent leur vie comme tous les autres enfants normaux. Néanmoins, ceux qui ont de crises sévères ou à répétition, connaissent souvent le retard ou l'abandon scolaire. Ces sujets drépanocytaires avec une symptomatologie sévère ne trouvent pas leur place dans la communauté. Cette attitude conduit à la stigmatisation et à la marginalisation. Ces enfants sont qualifiés de sorciers à la suite des différentes crises et des complications liées à la maladie peu comprise dans la communauté. Ce comportement de la population amène souvent les drépanocytaires adolescents au déni de la maladie et à l'abandon de soins afin de préserver leur dignité dans cette communauté. Les propos de ces parents illustrent cette constatation: « Souvent on l'insulte dans le quartier, même à l’école. Pendant qu'il passe en route, ses amis commencent à l'insulter d ‘enfant SS. C'est pour cette raison que son grand-père est allé le signaler à l’école pour qu'on ne le frappe plus. Juste au moment où cette information était donné à l’école, on se moque plus de lui. C'est ainsi qu'il m'a dit lorsque nous sommes arrivés ici à l'hôpital de ne pas dire qu'il est SS » (Madame Nz.)

#### Au sein de la famille

La présence de l'enfant drépanocytaire dans la famille entraîne des conséquences importantes. Les parents interrogés sur leur vécu quotidien disent que la maladie modifie l'ambiance familiale. Bien que la tendance générale soit de divorcer pour éviter de donner naissance à d'autres enfants drépanocytaires, la plupart de parents interviewés n'ont pas cédé aux pressions de membres de la communauté en général et de leur famille en particulier. Ainsi, la drépanocytose reste cachée à l'entourage familial; les parents gèrent seuls la situation pendant ou en dehors des crises de telle sorte que l'information ne soit pas diffusée au sein de la communauté. Dans certains cas, les parents ont tendance à surprotéger leur enfant malade et à manifester plus d'attention qu'aux autres membres de la famille. Les frères et saeurs du malade ont tendance à condamner le malade à cause de ses multiples crises qui déséquilibrent le budget familial en entraînant un coût important de soins de santé. « C'est pénible, comme j'ai accueilli mes nièces chez moi, chaque fois qu'il tombe malade, elles se moquent de lui. Elles disent: pourquoi seulement toi qui souffre à tout moment, tu fais dépenser l'argent de notre maman » (Madame Od.)

#### Au couple-parents

Les interrogations et les inquiétudes concernant l'avenir de l'enfant créent des tensions familiales, et par conséquent altèrent la qualité de vie du couple. Certains parents ont nié d'abord la maladie, ce qui fait qu'en dépit de l'annonce, ils se sont obstinés à ne pas fréquenter les structures de prise en charge rendant la survenue des certaines complications et la mort plus probables. Du point de vue économique, nos enquêtés disposent de revenus modestes, la plupart n'ont pas de professions rémunérées. La situation la plus préoccupante que nous avons rencontrée est celle d'une mère retenue avec sa fille en hospitalisation pour le non-paiement de la facture de 50 dollars américains. Un jour après, son enfant a rechuté entrainant la prolongation du séjour à l'hôpital et en conséquence l'augmentation du montant de la facture. Outre ces répercussions, nous avons aussi retenu que l'angoisse de parents reste permanente, exacerbée par des crises vaso-occlusives fréquentes, imprévisibles et souvent de survenue brutale surtout chez le malade. Les crises sont souvent brutales et imprévisible. Ces crises nécessitent des hospitalisations fréquentes entraînant l'augmentation des dépenses et la restriction des activités professionnelles des parents et donc un manque à gagner financier pour la famille.

#### Après le décès de l'enfant suite à la drépanocytose

Ces entretiens ne concernaient que les parents qui avaient déjà perdu un enfant drépanocytaire et qui venaient au centre pour le suivi du second enfant drépanocytaire; au total 3 parents dans cette situation. Ces parents ressentent un épuisement physique et psychique. Ils cherchent à comprendre les raisons de cette situation dramatique dans leur vie. Cette première expérience renforce leurs sentiments de culpabilité et augmente leur isolement, telle la mère qui a rompu les relations avec sa famille qui voudrait à tout prix mettre fin à son mariage après la mort de son fils. Voici ce qu'elle dit: « je n'ai plus de contact avec ma famille parce que mes frères voulaient que je divorce avec mon mari après la mort de notre fils. Comme j'avais refusé, tout le monde m'a abandonné ». Ce moment du deuil est un moment ou le soutien psychologique s'avère indispensable pour continuer à s'occuper des autres enfants surtout s'il y a d'autres drépanocytaires.

#### Perception de la prise en charge

Tous nos enquêtés semblent dire que la prise en charge n'est pas globale. Elle est essentiellement focalisée sur les aspects médicaux de la maladie. Les aspects socio-culturels et psychologiques qu'engendrent la maladie ne sont pas pris en compte au CMMASS. En dehors des médecins et des infirmiers, aucune autre institution ou profession n'intervient dans la prise en charge. Comme les soins coûtent chers et qu'il n'existe aucun financement, les parents sont enclin à l'automédication et n'amènent les enfants au centre que lorsque la situation devient critique: « Lorsque l'enfant fait la crise, j'achète moi-même certains médicaments que je connais, je lui donne et ça passe. Au cas contraire, s'il n'y a pas de changement, surtout si son abdomen reste distendu, je suis obligée de l'amener ici. Là on fait les examens et on prescrit les médicaments » (Madame Ang).

#### Besoins et problèmes liés à la prise en charge et quelques solutions proposes

La participation communautaire dans la prise en charge de leur santé commence par l'identification par eux-mêmes de leurs problèmes et de ressources pour les résoudre. Le centre fonctionne sans subside, il n'est pas équipé et tous les enquêtés avaient confirmé la vétusté du centre. Pour les parents, les problèmes résident dans la qualité de soins et le coût de la prise en charge de la maladie qui ne leur permet pas un suivi régulier de leurs enfants. Les conditions de vie difficile à Kinshasa exposent les drépanocytaires à plusieurs facteurs favorisant les crises, notamment, la difficulté d'accès à l'eau potable, le climat chaud et humide, l'insalubrité publique, la non observance du traitement suite au déni de la maladie et à l'insuffisance des ressources financières pour le suivi médical du malade en absence d'un système de sécurité sociale dans le système de santé en RDC. Pour améliorer la prise en charge, les parents proposent l'implication du gouvernement dans le financement de soins de santé de leurs enfants au même titre que le paludisme, la tuberculose et le VIH/sida. A titre d'exemple voici l'extrait de certains parents à ce sujet: « la prise en charge de ma famille est possible seulement si nous avons de moyens. Il arrive de moments où nous n'en disposons pas. Si les soins étaient presque gratuits comme on les donne aux tuberculeux, ça nous soulagerait ».

## Discussion

Ce travail est à notre connaissance la première étude qualitative sur la drépanocytose en Afrique centrale. Quelques études ont été menées sur le sujet au Nigeria en Afrique de l'Ouest [25, 26] et au Kenya en Afrique de l'est [27]. Cette étude qualitative a été menée au CMMASS de Kinshasa, première structure hospitalière créée en Afrique Centrale pour la prise en charge des patients drépanocytaire. C'est un centre de soins mixtes associant la médecine moderne à la médecine traditionnelle et la recherche. Le choix de ce centre a été motivé par des considérations épidémiologiques et cliniques, ainsi que par des raisons pratiques dans la réalisation de l’étude. Les parents qui le fréquentent constituent, de ce fait, une population qui se prête le mieux à cette étude.

Lors de notre recherche, les pères étaient souvent absents. Chez nos participants, lorsque l'enfant est malade, c'est la mère qui est souvent présente à l′hôpital, pendant que le père est parti soit au travail, soit dans la recherche du soutien matériel auprès de ses proches pour payer les soins de l'enfant. La présence de l′enfant malade dans la famille a une influence négative sur les relations interpersonnelles. Vivre avec un enfant qui a une maladie chronique est une expérience qui demande beaucoup de patience et des ressources de la part des parents. La réaction de panique de parents face à l'annonce de la maladie est plus liée à la méconnaissance de la drépanocytose. Le déni de la maladie et le silence que les parents gardent témoignent du caractère dévalorisant de la drépanocytose dans la société Africaine [25–27]. Ces expériences ont été rapportées dans d'autres études africaines [19–22, 27]. A la lumière des propos de nos enquêtés, la drépanocytose constitue une charge financière lourde pour les familles. Ils ont pris l'habitude de n'amener les enfants à l'hôpital que lorsque la situation devient grave et/ou après avoir réuni des moyens financiers pouvant leur permettre d'assumer les premiers soins. Les parents évoluent dans un environnement de pauvreté en absence de sécurité sociale et la méconnaissance de la gestion des crises à domicile (pauvreté et drépanocytose). Ces crises drépanocytaires entraînent de dépenses familiales imprévues. Pour pallier à cette situation de dépenses exagérées de soins, Hamza et al. en Tunisie avait proposé dans leur étude l'octroi aux patients d'une carte de handicap pour soulager certaines familles sur le plan financiers [28]. La gratuité ou la subvention des soins pour cette catégorie des patients et l’élaboration d'un projet de microfinances pour les familles pourraient permettre à moyen terme aux familles de faire face à certaines dépenses mais aussi à assurer un suivi plus régulier du patient.

La création des unités de prise en charge de la drépanocytose dans les différents niveaux du système de santé Congolais et leur insertion dans les soins intégrés pourraient aussi faciliter l'accès des malades aux soins en évitant de longues distances dans un système où les urgences ambulatoires ne sont pas assurées. Il n'existe, à ce jour, aucun circuit d'approvisionnement en médicaments essentiels pour les drépanocytaires. Les malades sont obligés ainsi de payer de leur propre poche ces médicaments généralement prescrits ad vitam [29]. Selon les propos recueillis auprès de nos enquêtés, il ressort que certains d'entre eux préféraient passer par l’église ou par la médecine traditionnelle au lieu d'amener l'enfant à l'hôpital. La répétition des crises, l'ignorance de la maladie, la pauvreté et le sentiment d'impuissance de la médecine occidentale peuvent en effet pousser les parents à recourir à des médecines parallèles comme l'ont montré des études antérieures dans notre milieu [30, 31].

La gestion de la maladie est possible à partir du moment où la communauté nationale met en place les actions nécessaires pour réduire son incidence à la naissance mais aussi la morbi-mortalité liée aux différentes crises notamment par des campagnes d'information, d’éducation et de sensibilisation. La participation communautaire aide à orienter les activités vers la promotion de la santé en impliquant un effort de chacun de ses membres [32–34].

## Conclusion

Cette étude a porté sur l'analyse des répercussions psychosociales de la drépanocytose chez les parents et proches La drépanocytose reste une maladie taboue. Les problèmes étant identifiés par nos enquêtés, il s'avère que les ressources du CMMASS sont limitées et que la prise en charge globale et de qualité est impossible. L'implication de l'Etat et des organismes humanitaires est importante pour soulager tant soit peu les parents en finançant les actions communautaires, en impliquant la participation de toute la population dans la lutte contre la drépanocytose.
